# Supplementary material for: Causal Relationship between Aging and Anorexia Nervosa: A White-Matter-Microstructure-Mediated Mendelian Randomization Analysis
Source: Biomedicines. 2024 Aug 16;12(8):1874. doi: 10.3390/biomedicines12081874 (PMC11351342; doi:10.3390/biomedicines12081874)
Supplement: Supplementary file 1 [file biomedicines-12-01874-s001.zip › Supplementary Figure/Figure S4/Singe SNP plot of MR analysis for TL to ubm-b-1990.pdf]

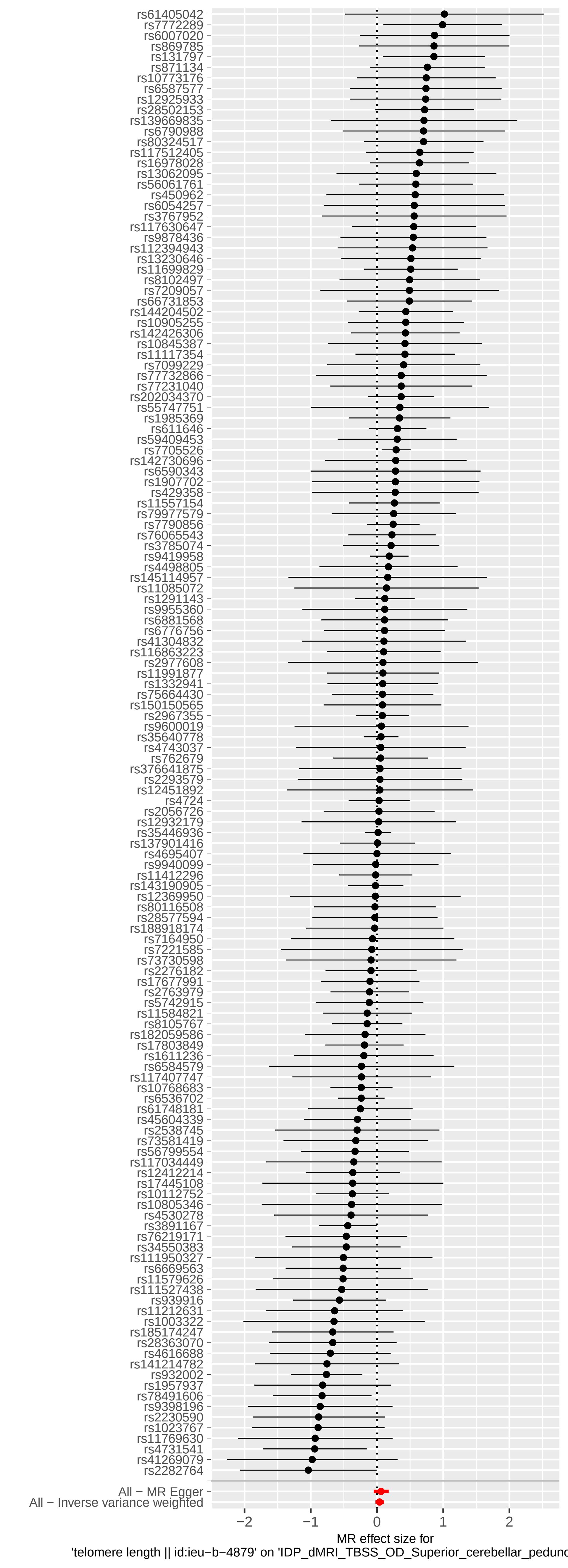
rs61405042
rs7772289
rs6007020
rs869785
rs131797
rs871134
rs10773176
rs6587577
rs12925933
rs28502153
rs139669835
rs6790988
rs80324517
rs117512405
rs16978028
rs13062095
rs56061761
rs450962
rs6054257
rs3767952
rs117630647
rs9878436
rs112394943
rs13230646
rs11699829
rs8102497
rs7209057
rs66731853
rs144204502
rs10905255
rs142426306
rs10845387
rs11117354
rs7099229
rs77732866
rs77231040
rs202034370
rs55747751
rs1985369
rs611646
rs59409453
rs7705526
rs142730696
rs6590343
rs1907702
rs429358
rs11557154
rs79977579
rs7790856
rs76065543
rs3785074
rs9419958
rs4498805
rs145114957
rs11085072
rs1291143
rs9955360
rs6881568
rs6776756
rs41304832
rs116863223
rs2977608
rs11991877
rs1332941
rs75664430
rs150150565
rs2967355
rs9600019
rs35640778
rs4743037
rs762679
rs376641875
rs2293579
rs12451892
rs4724
rs2056726
rs12932179
rs35446936
rs137901416
rs4695407
rs9940099
rs11412296
rs143190905
rs12369950
rs80116508
rs28577594
rs188918174
rs7164950
rs7221585
rs73730598
rs2276182
rs17677991
rs2763979
rs5742915
rs11584821
rs8105767
rs182059586
rs17803849
rs1611236
rs6584579
rs117407747
rs10768683
rs6536702
rs61748181
rs45604339
rs2538745
rs73581419
rs56799554
rs117034449
rs12412214
rs17445108
rs10112752
rs10805346
rs4530278
rs3891167
rs76219171
rs34550383
rs111950327
rs6669563
rs11579626
rs111527438
rs939916
rs11212631
rs1003322
rs185174247
rs28363070
rs4616688
rs141214782
rs932002
rs1957937
rs78491606
rs9398196
rs2230590
rs1023767
rs11769630
rs4731541
rs41269079
rs2282764
All − MR Egger
All − Inverse variance weighted
−2
−1
0
1
2
MR effect size for
'telomere length || id:ieu−b−4879' on 'IDP_dMRI_TBSS_OD_Superior_cerebellar_pedunc
